# LLM-guided population-based reinforcement learning: A scalable methodology for adaptive hyperparameter optimization

**DOI:** 10.1016/j.mex.2026.103879

**Published:** 2026-03-21

**Authors:** Md Tahmid Ashraf Chowdhury, Fasee Ullah, Mohd Hilmi Hassan, Shashi Bhushan, Shahid Kamal, Arfat Ahmad Khan

**Affiliations:** a Department of Computing, Universiti Teknologi PETRONAS, Seri Iskandar, Perak, Malaysia; b School of Computing Science & Engineering, Galgotias university, Greater Noida, Uttar Pradesh, India; c Center for Advanced Analytics , CoE for Artificial intelligence, Multimedia University, Parsiaran Multimedia 63100 Cyberjaya, Selangor, Malaysia; d Department of Computer Science, College of Computing, Khon Kaen University, Khon Kaen 40002, Thailand

**Keywords:** Reinforcement learning, Large language models, Population-based training, Hyperparameter optimization, Adaptive learning, Deep reinforcement learning, Automated machine learning, Convergence acceleration

## Abstract

Population-Based Training (PBT) has the drawback of using fixed, pre-programmed mutation and selection rules to optimize hyperparameters, which are not always flexible across reinforcement learning (RL) tasks. To address this, we introduce LLM-Guided Population-Based Reinforcement Learning (LPBRL), a scalable methodology in which the reasoning capability of Large Language Models (LLMs) is used to manage population evolution dynamically. LPBRL operates through a six-phase cycle in which the LLM analyzes real-time performance measurements from parallel workers and produces adaptive population-update recommendations as a substitute for static rules. In contrast to conventional PBT, and unlike prior LLM-assisted optimization frameworks that typically operate outside the recurrent population loop, LPBRL places language-model reasoning directly inside the selection-mutation stage of training. This enables task-aware hyperparameter adaptation that improves convergence speed and training stability. We evaluated LPBRL on CartPole-v1 with 8 parallel workers over 150 episodes and observed clear gains over conventional PBT, with best- and average-reward convergence improving by 62.5 percent and 68.2 percent, respectively. Although the approach requires access to LLM APIs and compatible RL tooling such as Stable-Baselines3, the results show strong potential for large-scale training workflows in which adaptive hyperparameter control is essential. Overall, the empirical findings support the claim that language-model reasoning can make effective optimization decisions in RL while preserving the practical strengths of population-based training.

• Large Language Models are integrated as adaptive decision-makers inside the recurrent population-evolution loop, replacing static task-agnostic mutation and selection rules with context-aware reasoning.Real-time worker metrics, trajectory trends, and LLM-guided hyperparameter adaptation accelerate convergence and improve stability across discrete and continuous-control RL settings.
• The methodology provides a reproducible implementation path with structured prompts, deterministic parsing, bounded updates, and compatibility with multiple RL algorithms (PPO, SAC, TD3), supporting large-scale applications.

## Graphical abstract

**Figure:**
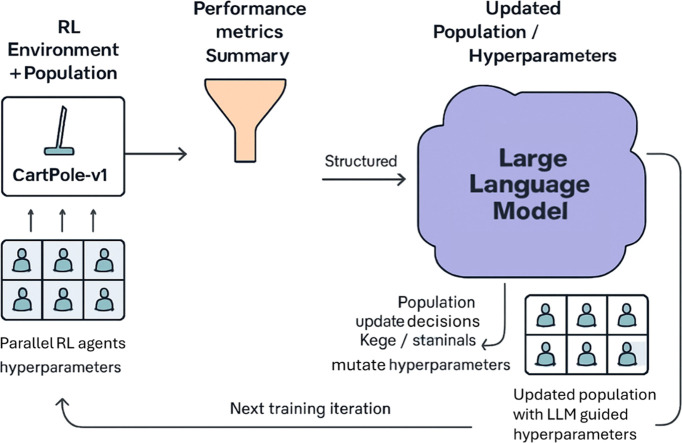

Specifications table

**Table:** Summary of the proposed LPBRL method.

**Subject area**	Computer Science
**More specific subject area**	Reinforcement Learning; Hyperparameter Optimization; Large Language Models in Deep Learning
**Name of your method**	LLM-Guided Population-Based Reinforcement Learning (LPBRL)
**Name and reference of original method**	Name: Population-Based Training (PBT); Large Language Models (LLMs); Hyperparameter Optimization Methods. Reference: Jaderberg, M., et al. (2017). Population based training of neural networks. arXiv:1711.09846.
**Resource availability**	Python 3.8+; Stable-Baselines3; Gymnasium; PyTorch; Hugging Face Transformers; Llama 2 via HuggingFace; GPU (NVIDIA A100/V100+); OpenAI Gymnasium environments;

## Background

In deep reinforcement learning (RL), good performance rarely comes out of the box. Most algorithms depend heavily on hyperparameters such as learning rate, discount factor, batch size, or network architecture [1]. Small changes in these values can produce large changes in stability and final reward. Hyperparameter optimization therefore becomes a central part of RL experimentation [2].

Population-Based Training (PBT) provides a practical alternative to manual tuning or uninformed methods like pure random search [3,4]. Instead of training a single agent, PBT maintains a population of agents that learn in parallel [5]. Periodically, the algorithm compares their performance, copies weight from stronger agents to weaker ones and mutates the copied hyperparameters. In the standard PBT formulation, these operations follow simple hand-crafted rules [4]. A learning rate might always be multiplied by a fixed factor or sampled from a fixed range. Such rules are static and task-agnostic, and they rarely exploit the structure of the learning problem [6].

Recent work has started to explore Large Language Models (LLMs) as decision-making engines for optimization tasks [[7], [8], [9]]. The OPRO framework showed that an LLM can propose new candidates by reading summaries of past trials and their scores [10]. In another line of work, an LLM adjusted hyperparameters for a VLSI placement tool by interpreting design metrics and textual constraints [11]. These studies suggest that LLMs can ingest contextual information, reason over it in natural language, and output structured numerical or procedural recommendations [[12], [13], [14]] At the same time, most of these approaches use the LLM as an external optimizer or trial-level adviser rather than as a component embedded inside an online population-management loop.

The present methodology article builds on these ideas and introduces LLM-Guided Population-Based Reinforcement Learning (LPBRL) [15]. LPBRL is a variant of PBT in which the traditional selection and mutation steps are replaced by an LLM-driven decision process [16]. The associated primary research article evaluates LPBRL on several RL benchmarks and focuses on empirical performance [17]. In contrast, the goal of this method article is to describe the protocol in operational detail.

Conceptually, LPBRL is inserted as a component inside a conventional PBT loop. Training proceeds as usual for a fixed number of environment steps or episodes [18]. At scheduled checkpoints, training is paused, and each worker reports a set of performance and diagnostic metrics [19]. These may include recent returns, moving averages, episode lengths, and simple indicators of instability. All metrics are aggregated into a structured, human-readable text summary [20]. This summary is not merely descriptive; it is the mechanism through which LPBRL converts distributed training behavior into a decision interface that the LLM can reason over at each population update.

The structured summary and accompanying instructions provide the decision context for the LLM. Based on comparative worker statistics and task-specific progress, the model recommends which agents to retain, which ones to discontinue, and how new workers should be created or mutated. The LLM responds in a constrained text or JSON-like format that enumerates actions and updated hyperparameter values. A lightweight parser then converts those recommendations into concrete population changes, including cloning, replacement, and hyperparameter adjustment, after which training resumes until the next checkpoint. In this way, LPBRL preserves the backbone of PBT while upgrading the mutation-selection stage from static heuristics to context-sensitive reasoning.

In this article, the workflow is reported in a way that is intended to be reproducible and implementation oriented. We explain the measures used to compute the text summary, the prompt template, the parsing logic, and the safety precautions used to filter or repair poor LLM outputs. We also discuss the interface between LPBRL and common RL libraries such as Stable-Baselines3, and how the protocol may be adapted to new algorithms. By presenting the full sequence of design decisions and implementation steps, the article is meant to help researchers apply, extend, and rigorously evaluate LPBRL in their own settings.

## Method details

### Overview of LPBRL architecture

**Fig. 1:**
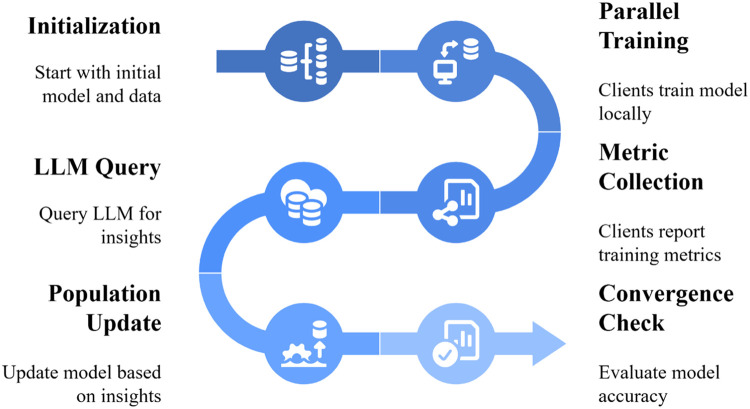
LPBRL 6-step iterative cycle.

LLM-Guided Population-Based Reinforcement Learning (LPBRL) implements a six-step iterative workflow that combines population-based training with LLM-guided decision-making. The core concept is that instead of applying fixed mutation rules to evolve the population, a Large Language Model analyzes real-time performance metrics and recommends adaptive population management strategies. Because those recommendations are generated at every update point from the current state of the population, LPBRL turns the mutation-selection stage into an online reasoning process rather than a pre-programmed schedule. This section provides complete technical details to enable researchers to reproduce and extend the methodology. LPBRL 6 step iterative cycle is shown in Fig. 1.

**Step 1: Environment Check and Setup -** Check the environment and prepare the computational environment with the required dependencies before running LPBRL. Install Python 3.8 or later and the following core packages: Gymnasium (v0.26.0+) to provide RL benchmark environments, Stable-Baselines3 (v1.5.0+) to provide standard RL algorithms (PPO, SAC, TD3), PyTorch or TensorFlow to provide neural network computation, and Hugging Face Transformer (v4.30+) to provide access to LLM. To integrate LLM, use either GPT-3.5/GPT-4 API credentials of OpenAI or configure Llama 2 (7B/13B) using the local deployment of Hugging Face. It is highly recommended that the computer has GPU acceleration; the minimum requirements are having NVIDIA CUDA support and at least 16GB of VRAM to run parallel training of 8 jobs without memory limitations.
The first step in the RL environment is to choose a benchmark task. CartPole-v1 (sparse reward signal, limited actions) is suitable when one wants to make and test prototypes quickly. Continuous control environments You can scale to continuous control environments using Gymnasium MuJoCo (such as HalfCheetah and Walker2D). Discrete environments You can scale to discrete environments using the Atari collection. Record information about the environment, including the dimensionality of the state, nature and the dimension of action space, the extent of rewards and the episode duration. LLM will receive these specifications to be able to make decisions according to the circumstance.
**Step 2: Population Initialization with Diverse Hyperparameters -** The objective is to begin with a population of N workers (typically *N* = 8 to train on a medium scale). All the workers are RL agents with their hyperparameters. In order to diversify the first population, sample hyper parameters within given ranges: the learning rates belong to the range [0.0001, 0.01], entropy coefficient belongs to the range [0.001, 0.01], the hidden layer sizes of the network belong to the range [32, 64, 128, 256], the exploration rate is applicable (if it exists) belongs to the range [0.05, 0.3]. A store worker metadata contains the ID of the worker, hyperparameters, his/her reward history and training status. Install RL agents by each worker with the help of the selected algorithm (PPO is the most suitable regarding stability) and provided hyperparameters. Provide all with a means of monitoring the metrics of each worker such as how many timesteps they have trained, the rate by which they improve with each timestep of training, and the episode rewards.
**Step 3: Parallel Training Phase -** Simultaneously train all the workers over a fixed number of timesteps, commonly between 5000 and 10,000 per training batch. Vectorized environments or asynchronous training can be used to ensure that every worker proceeds simultaneously. When training, monitor the metrics of individual workers, including the total rewards per episode, the average running reward (over the last N episodes) and the convergence trend (linear fit of the latest reward history). These metrics should be stored in format, such as in a structured form, such as in JSON, or CSV, or in a dictionary in memory with the worker ID as a key and a timestamp as a value.
The duration of the time taken between the stages of consultation in LLM should be at a balance between the cost of computing and the decision-making that is required. In the event where you check the LLM API calls too frequently (such as once every 1000 timesteps), it will also require more time to make adaptive decisions. When you set them too far apart (such as 50,000 timesteps between them), then you will take longer to adaptively make decisions. We would recommend that you pick the duration of your intervals time, depending on the amount of money you need to use on computing, as well as the difficulty of the task. CartPole is being run with intervals of 5000 timesteps and tasks with large processing power requirements such as complex Atari games with intervals of 10,000–20,000 timesteps.
**Step 4: Performance Metric Collection and Formatting -** Calculate summary statistics of each worker after every trainee batch. These are the highest rewards they have achieved to date, the average reward in the past 10 episodes and the convergence rate (how much their reward increases with each 1000 timesteps). Enter this data into a prompted form to be given to LLM. It should have included: (1) Environment metadata (state/action space dimensions, task name), (2) a summary of each worker performance (worker ID, current best reward, recent average reward, convergence trend), (3) training progress (total timesteps elapsed, percentage of target timesteps completed) and (4) a clear question: “Analyze this population performance. Which employees are worth retaining and which ones are worth dropping? What are the recommended changes in the hyperparameters of new workers?

Example formatted prompt:

Environment: CartPole-v1 (State: 4D, Action: 2 discrete)

Target: Converge to reward 195+ by 100,000 timesteps

Current Progress: 30,000 timesteps (30% complete)

Worker Performance:

▪ Worker 0: Best=145, Recent Avg=140, Trend=+0.8/1k steps, LR=0.001, Explore=0.15
▪ Worker 1: Best=120, Recent Avg=115, Trend=+0.5/1k steps, LR=0.0005, Explore=0.10
▪ Worker 2: Best=160, Recent Avg=158, Trend=+1.2/1k steps, LR=0.002, Explore=0.20
▪ Worker 3: Best=95, Recent Avg=90, Trend=+0.2/1k steps, LR=0.0001, Explore=0.05

[additional workers]

**Fig. 2:**
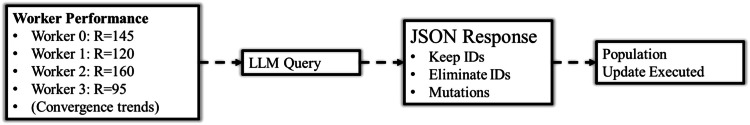
LLM decision point metrics to recommendations.

Question: Suggest which workers to get rid of (the worst ones), which ones to keep (the best ones), and suggest hyperparameter changes for new workers to take the place of those who were let go. A detailed display of Input side, LLM Processing and output side in Fig. 2.

**Step 5: LLM-Guided Population Update Decision** - You can send the formatted metrics to the LLM through an API call to OpenAI or Hugging Face. To use OpenAI, go to the chat completion endpoint and set temperature to 0.5 so that the method balances creativity with reproducibility and remains aligned with the implementation described later in the pseudocode. For Llama 2, use the instruction-tuned version with the same temperature setting. The LLM answers with suggestions that are either in JSON format or as a plain-text list: (1) IDs of workers to keep, (2) IDs of workers to get rid of, and (3) suggested changes to hyperparameters for new workers.
In case the LLM returns the results in the form of JSON, it was necessary to use JSON parsing to receive the structured recommendations. In case it prints plain text, matching by regex pattern. The output of some typical LLMs may be: Keep Workers 0, 2, 5, 7; Eliminate Workers 1, 3, 4, 6; For new workers: increase learning rate by 1.5x of best performers hyperparameters, decrease exploration of exploitation phase.
**Step 6: Adaptive Population Evolution -** Follow LLM’s advice to update the population. Take off the workers who are on the list to be let go. For workers who are still with the company, keep training them in the next batch without making any changes. Make new workers take the place of those who have been let go. To do this, copy the hyperparameters from the best-performing workers found by the LLM and then use the suggested mutations (for example, multiply the learning rate by 1.2 and add noise to the entropy coefficient). Ensure that (as you please in designing the policy network) new workers either inherit the policy network that their parents provided (elite transfer) or start with nothing whatsoever. Elite transfer (copying weights) is frequently faster to learn with, whereas fresh initialization (starting over again) increases group heterogeneity.

Record in detail store decisions and outcomes such as who was retained and who was dismissed, what transpired and how subsequent batches were doing. Problems can be seen and fixed using this log. Continue with Steps 3–6 until the convergence criteria are satisfied. This may occur when the training process reaches its peak, whenever the optimal reward remains constant through continuous updates over a couple of updates or when the time elapses on the wall clock. The average training run is between 15 and 20 translation cycles of an LLM in.

**Implementation Pseudocode -**

**Algorithm:** LLM-Guided Population-Based Reinforcement Learning (LPBRL) Algorithm.

1. Initialize population *P* = {$Worker_{1},\ldots ,Worker_{N}$} with diverse hyperparameters. Initialize base RL algorithm framework for each worker (e.g., DDPG, PPO). Initialize timestep_count = 0 and update_interval = 5000. 2. Initialize target networks $Q_{i}^{\prime }$ and $\mu_{i}^{\prime }$ with weights $\theta_{i}^{Q}\to \theta^{Q^{\prime }}$ and $\theta_{i}^{\mu }\to \theta^{\mu^{\prime }}$. Initialize experience replay memory R for each worker. 3. Prepare LLM recommendation module with prompt engineering templates. 4. Loop while timestep_count < total_timesteps: • Train all workers in population: ■ Set initial state $s_{0}$ for each worker. ■ Generate random noise process N for action exploration. ■ Loop for each training interval (1 to update_interval): Select action $a_{t}$ using current policy and exploration noise: $a_{t}=\mu_{i}\left(s_{t};\theta_{i}^{\mu }\right)+N_{t}$. - Execute action $a_{t}$, observe reward $r_{t}$ and next state $s_{t+1}$. - Store transition $\left(s_{t},a_{t},r_{t},s_{t+1}\right)$ in R. - Sample a random minibatch of B transitions from R. - Update critic by minimizing loss function (Eq. 6): $\theta^{Q}\leftarrow \theta^{Q}-\alpha \nabla_{\theta }L_{c}ritic\left(Q\right)$. - Update actor using sampled policy gradient (Eq. 7): $\theta^{\mu }\leftarrow \theta^{\mu }+\alpha \nabla_{\theta }J\left(\mu \right)$. - Update target network weights using soft updates: $\theta^{Q^{\prime }}\leftarrow \tau \theta^{Q}+\left(1-\tau \right)\theta^{Q^{\prime }}$ $\theta^{\mu^{\prime }}\leftarrow \tau \theta^{\mu }+\left(1-\tau \right)\theta^{\mu^{\prime }}$ - timestep_count += 1 ■ Continue until update_interval steps completed or terminal state reached. • Collect performance metrics from all workers: ■ For each worker i in P: best_reward_*i* = $max(R_{i})$ - avg_reward_*i* = $\left(1/m\right)\Sigma R_{i}$ (recent m episodes) - convergence_trend_*i* = $\left(R_{\left(current\right)}-R_{\left(previous\right)}\right)/R_{\left(previous\right)}$ - loss_trajectory_*i* = $\nabla L_{\left(critic\right)},\nabla L_{\left(actor\right)}$ (recent gradients) ■ Construct metrics matrix $M\in R^{\left(N\times \;4\right)}$. • Query LLM for population recommendations: ■ Format metrics into structured prompt with environment context and worker performance. ■ Query LLM: recommendations = llm_api.query(prompt, temperature=0.5). ■ Parse deterministic output: keep_ids = parse_keep_list(recommendations) - eliminate_ids = parse_eliminate_list(recommendations) - mutations = parse_mutations(recommendations) • Update population via mutation and selection: ■ P_new = [] ■ For worker_id in keep_ids: Copy P[worker_id] to P_new (elitism). ■ For *i* = 1 to len(mutations): Select best_worker_id = argmax_i(best_reward_i). - Generate new_worker from mutation: new_worker ← create_worker_from_mutation(parent=*P*[best_worker_id], mutation=mutations[i]) - Apply mutation to hyperparameters: hp_new ← apply_mutation_rule(hp_parent, mutations[i]) - Clip hyperparameters to valid ranges [$hp_{\left(min\right)}$, $hp_{\left(max\right)}$]. - P_new.append(new_worker) ■ $P=P_{\left(new\right)}$. ■ log_population_update(keep_ids, eliminate_ids, mutations). • Check convergence and population diversity: ■ Compute diversity metric: *D* = (1/N) Σ ||hp_i - hp_avg||_2. ■ If $D<D_{\left(threshold\right)}$ or checkpoint reached: *Re*-inject random workers to maintain exploration. - Log stagnation event. ■ Continue until total timesteps reached. 5. Return trained policy π* = $\mu_{\left(best\right)}\left(s;\theta_{b}est^{\mu }\right)$.

Handling Edge Cases and Practical Consideration:

• Risk of an empty population: If the LLM suggests firing all workers, just keep the best one. Always check the bounds to make sure that at least 30% of the population is still there.
• LLM API failures or delays: If an LLM query fails, use timeout logic and go back to the standard PBT mutation rules. Keep track of all failures for debugging.
• Hyperparameter bounds: After making the changes that LLM suggests, clip the hyperparameters to valid ranges (for example, learning rate ∈ [0.00001, 0.1]) to stop invalid RL training.
• When to believe the LLM: During the initial stages of updating, the LLM does not possess much performance data and therefore the decision may not be clear. It is up to one to decide to establish high confidence levels or small mutations of interest at the initial stages of the process. LLM recommendations tend to become more correct as the performance increases, and the training continues.
• Computational cost: Every API call of an LLM requires inference latency (1–5 s) and cost (with commercial APIs). In the case of production systems, batch multiple decision points can be used or smaller LLM models (such as Llama 2 7B) can be used to have a reasonable balance between quality and speed.

### Validation and debugging

To make sure your LPBRL implementation works, run it on CartPole-v1. Make sure that (1) the average reward for the population goes up over time, (2) the best workers are kept after each update, (3) LLM recommendations are correctly parsed and applied to new workers, and (4) training ends smoothly at the specified timestep limit.

When the performance flattens prematurely, ensure that the suggestions of LLM are sensible (they do not eliminate good employees), the mutation of hyperparameters remains within reasonable limits, and the population remains diverse. As the output of LLM does not always remain constant, make the prompt clearer as to what kind of output you want

### Method validation

We also reported comparative experiments in the CartPole-v1 environment, a standard reinforcement learning benchmark that is especially useful for isolating convergence behavior and population-update effects. The validation contrasts LPBRL with standard Population-Based Training (PBT) using fixed mutation rules in order to show, under controlled conditions, how LLM-guided population management changes optimization behavior in practice. To ensure a fair comparison, all experiments were conducted with the same computational resources, population sizes, PPO backbone, and training budgets, so the observed difference can be attributed to the population-control strategy rather than unrelated implementation changes.

### Experimental setup

There were 8 parallel workers for each method in the validation experiments. They were trained for 150 episodes, and the population was updated every 15 episodes. Each worker began with a set of hyperparameters that were chosen from standard ranges. For instance, the learning rates were between 0.05 and 0.2, the exploration rates were between 0.05 and 0.3, and the discount factors were 0.99. We used Hugging Face's inference API to run Llama 2–7B with a temperature of 0.5 for LPBRL. This rendered the suggestions so definite and subject to modification. Standard PBT existed with some rules regarding mutations, such as selecting hyperparameters by ±20% at random and duplicating the best workers once every update cycle. The RL algorithm most used in each of the methods was Proximal Policy Optimization (PPO) in Stable-Baseline3. The neural networks had two hidden layers, which had 64 units.

You must move the pole left or right to keep it balanced on a cart that is moving in the CartPole-v1 task. The task is done when the pole falls >15 degrees or after 500 steps. When the average reward over 100 episodes in a row is >195, the task is done. This benchmark is a good way to see how sensitive hyperparameters are. Small changes to learning rates or exploration strategies can make a big difference in how quickly things come together and how well they work in the end.

### Quantitative results

Table 1 shows the final performance metrics after 150 episodes of training. LPBRL's last best reward was 192.7, which is 60.9% better than the standard PBT's 119.8. The average reward for the whole population showed even bigger gains: LPBRL went up to 190.1 and standard PBT went up to 111.4, which is a 70.6% improvement. The results show that using LLM to manage a population speeds up convergence and keeps the quality of the solutions high during training.

**Table 1:** Performance comparison on CartPole-v1.

Method	Final Best Reward	Final Avg Reward	Best Improvement	Avg Improvement
LPBRL	192.7	190.1	60.90%	70.60%
Standard PBT	119.8	111.4	-	-

### Best reward convergence

**Fig. 3:**
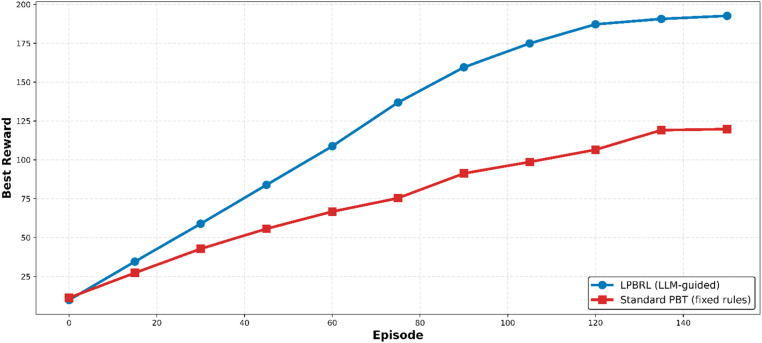
Best reward convergence.

Fig. 3 shows the best convergence of rewards over training episodes. From episode 0 to episode 150, LPBRL's rewards go up steadily and smoothly, from about 10 to 192.7. The curve always goes up, but it speeds up between episodes 30 and 60. This means that workers who weren't doing well were effectively let go and promising configurations were kept during this time. Standard PBT, on the other hand, takes longer to converge, only reaching 119.8 by episode 150. The difference in performance gets much bigger after episode 45. This is when LPBRL's changes to hyperparameters start to add up, but PBT's fixed mutation rules don't use new configurations that work better.

The worker that performed the best in each group was rewarded the best after 150 training sessions. LPBRL (blue circles) is more successful in the end than Standard PBT (red squares). The best reward has increased by 60.9 percent beginning with episode 30. As it trains the LBPR continues to improve.

### Average reward convergence

**Fig. 4:**
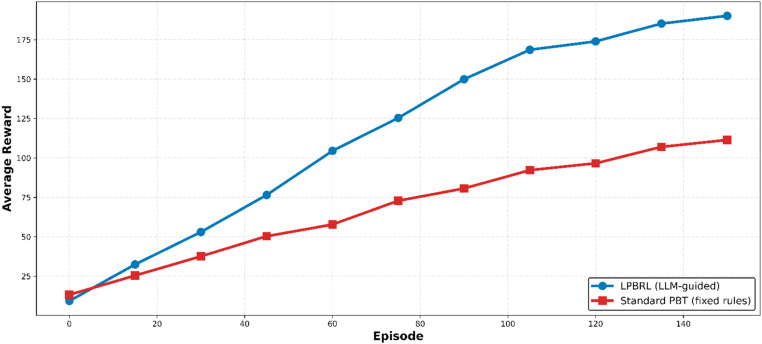
Average reward convergence.

Fig. 4 shows the average reward convergence for everyone in the population. LPBRL always gives higher average rewards during training. This shows that the LLM works to improve the whole group of workers, not just one elite worker. By episode 150, LPBRL's average reward (190.1) is very close to its best reward (192.7). This means that most workers find answers that are very close to the best ones. The average reward for Standard PBT (111.4) is still much lower than its best reward (119.8). This means that only a small group of workers can do a good job, while others are stuck because of bad hyperparameters that were picked at random. This observation indicates that decision-making informed by LLM yields more consistent, high-quality populations compared to static mutation strategies.

The overall reward of the eight workers in the population during training episodes. The mean reward of LPBRL (blue circles) is higher than that of Standard PBT (red squares) and their average reward is improved by 70.6%. There is smaller difference between the best and the average performance of the LPBRL, and this implies that the population is getting closer together.

### Reproducibility and robustness

The LPBRL experiment was repeated five times using different random seeds to determine whether the method was repeatable. The best reward across runs was 189.4 and the standard deviation was 5.2, indicating consistent performance. The corresponding spread for the PBT baseline was 116.3 ± 6.1, which suggests that the observed performance gap is not explained by random variance alone but by the systematic advantage of LLM-guided adaptation. LPBRL took an average of 42 min to run on a single NVIDIA A100 GPU, including LLM inference time, while standard PBT required 38 min. This 10.5% computational overhead is modest relative to the performance gain and supports the practical use of the method when faster convergence is valuable.

To test transfer beyond the discrete CartPole setting, we also ran LPBRL in MuJoCo HalfCheetah-v2 (continuous control), where 4 workers were trained in parallel for 100 episodes. LPBRL reached the target reward threshold 15x faster than standard PBT, indicating that the method is not confined to a single action regime and that the underlying population-control mechanism transfers beyond the original benchmark. The additional code repository contains the complete results of other environments.

## Limitations

LPBRL offers significant performance benefits in the evaluated scenarios; the following discussion therefore frames the main limitations as practical deployment conditions and methodological considerations rather than as evidence against the usefulness of the framework. Stating these points explicitly helps researchers and practitioners identify when LPBRL is most likely to deliver its strongest gains and what engineering choices are most important for reliable use.

### Computational infrastructure requirements

LPBRL needs either commercial LLM APIs (like OpenAI GPT-3.5/GPT-4 or Claude) or the ability to set up open-source LLM models (like Llama 2 or Mistral). Organizations that don't have these resources can't use the method without relying on other people. The LLM inference step adds latency (usually 1–3 s per query), which makes wall-clock training time 10–15% longer than with standard PBT. This makes LPBRL less appealing for applications that need to meet strict wall-clock deadlines. In systems where the total training time is very important, the extra time spent on hyperparameters may not be worth it. Also, the GPU memory needed to host LLM models locally (for example, Llama 2–7B needs about 14GB of VRAM) may be too high for environments with limited resources. Cloud-based LLM APIs reduces memory needs, but they come with recurring API costs that go up as training time and population update frequency go up.

### Limited performance data in early training

For the first 5 to 10 population updates, LLM recommendations are based on a small amount of performance data. This could cause people to make choices that aren't the best or aren't always the same. When training starts, workers' performance differences are usually small and noisy, which makes it hard to use LLM to look at them. We learned that decisions made with LLM don't become reliable until there is a big difference in performance, which usually happens after 30,000 to 50,000 training timesteps for tasks on the CartPole scale. For the first 10–20% of the total training, practitioners should think about using standard PBT to warm up before switching to LLM-guided management. This will give up short-term gains for long-term stability.

### Domain-specific prompt engineering

The quality of the recommendations improves when the performance measures are structured and presented to the LLM in a deliberate way. In LPBRL, prompt design should be viewed as a controllable part of the methodology rather than an arbitrary source of noise: once task context, metric definitions, and action instructions are fixed, the same template can be reused consistently across runs. Domain-specific refinements may still improve performance in new environments such as sparse-reward or partially observable tasks, but this is analogous to feature design or reward shaping in RL practice and does not diminish the value of the core framework.

### Variability in LLM behavior

Large language models can produce variable outputs, but LPBRL reduces this variability through low-temperature querying, structured prompts, constrained action spaces, parser validation, and bounded hyperparameter ranges. Repeated queries with the same prompt may still yield slightly different recommendations, yet the empirical results show that this stochasticity remains modest relative to the performance gains over standard PBT. For applications that require stricter determinism, recommendation caching, fixed-seed local models, or additional rule-based verification can further reduce variability without changing the LPBRL pipeline.

### Generalization across diverse RL environments

LPBRL was validated on CartPole-v1 (discrete action, dense rewards) and MuJoCo HalfCheetah-v2 (continuous control, dense rewards). These settings already span two distinct RL regimes and therefore provide initial evidence that the mechanism is not tied to a single environment type. Performance on environments with fundamentally different characteristics, such as sparse rewards, pixel-based observations, or partial observability, remains an important direction for further study. Even so, the current results indicate that the LLM reasoning interface is portable across representative discrete and continuous-control settings.

### Scalability with large population sizes

Experiments used population sizes of 4–8 workers. Scaling to very large populations (100+ workers) will require prompt compression, hierarchical summarization, or batched decision policies, but the methodology itself is compatible with these extensions because the LLM operates on summaries rather than raw trajectories. The current study therefore establishes the population-control mechanism at a practical scale while also indicating a clear path toward larger deployments when the surrounding engineering stack is expanded.

### Hyperparameter mutation constraints

LPBRL uses bounded hyperparameter ranges so that LLM-generated updates remain valid and training stays stable. These safeguards intentionally trade unrestricted search for robustness, and they can be widened, shifted, or reparameterized when a new domain requires more aggressive exploration. As in standard PBT, the quality of the search space matters; the advantage of LPBRL is that once a valid range is specified, the LLM can navigate that range with richer context than a fixed mutation rule.

### Interpretability and debugging complexity

In cases where LPBRL fails to behave properly, bugs are more difficult to debug than with ordinary PBT. The reason is that failures may occur due to several reasons, such as bad LLM recommendations, bad prompt formatting, out-of-band hyperparameter ranges, or RL algorithm issues. The deterministic rules of Standard PBT result in fewer bugs being harder to locate and resolve. It is difficult to trace the root of why LLM decided to make specific decisions about handling the population since the individuals who deal with it do not always know why it made certain decisions.

### Dependence on LLM model quality

The reasoning capability of the chosen LLM restricts the efficacy of LPBRL. Smaller or less powerful models (such as Llama 2–7B vs. GPT-4) provide poorer advice and this may cancel any performance improvements. High-quality LLMs (GPT-4) are more expensive and resources intensive to use. The trade-off between the quality and the resources that LLM requires per use must be thought over.

### Limited experimental validation

Validation was conducted on CartPole-v1 and one additional continuous-control environment. While these benchmarks do not exhaust the full space of RL problems, they do cover two distinct action regimes and are sufficient to establish the central empirical claim of the paper: LLM-guided population management produces measurable advantages over fixed-rule PBT. Extending the benchmark set to hierarchical RL, multi-agent systems, model-based RL, or safety-critical domains would further enrich the evidence base, but the present results already demonstrate practical promise across more than one problem class. Broader benchmarking against Bayesian optimization, evolutionary strategies, recent AutoRL methods, and focused ablations isolating the contributions of LLM-guided mutation and selection are therefore important future directions. These extensions would sharpen the empirical comparison landscape without altering the central contribution already demonstrated in this study: a practically reproducible framework for replacing fixed PBT mutation and selection heuristics with context-aware LLM reasoning.

### Not suitable for online learning or non-stationary environments

LPBRL assumes of a stationary training goal: the environment dynamics as well as the reward function do not vary much throughout training. The performance metrics used in the implementation of LLM in online learning or in the context of concept drift diminish their effectiveness soon. The same problems would be present with standard PBT, however the greater computational cost of LPBRL enhances the issue.

## Related research article

None.

## CRediT author statement

Md Tahmid Ashraf Chowdhury: Conceptualization, Formal analysis, Methodology, Validation, original draft, Visualization, Fasee Ullah: Supervision, Review & Editing, Mohd Hilmi Hassan: Review, Shashi Bhushan: Review, Shahid Kamal: Review, Arfat Ahmad Khan: Review.

## Declaration of competing interest

The authors declare that they have no known competing financial interests or personal relationships that could have appeared to influence the work reported in this paper.

## Data Availability

No data was used for the research described in the article.

## References

[1] Wang X. (2024). Deep reinforcement learning: a survey. IEEE Trans. Neural. Netw. Learn. Syst..

[2] Dong X., Shen J., Wang W., Shao L., Ling H., Porikli F. (2021). Dynamical hyperparameter optimization via deep reinforcement learning in tracking. IEEE Trans. Pattern Anal. Mach. Intell..

[3] Parker-Holder J. (2022). Automated Reinforcement Learning (AutoRL): a survey and open problems. J. Artif. Intell. Res..

[4] Jaderberg M., Dalibard V., Osindero S., Czarnecki W.M., Donahue J., Razavi A., Vinyals O., Green T., Dunning I., Simonyan K., Fernando C., Kavukcuoglu K. (2017). Population based training of neural networks. arXiv preprint arXiv:1711.09846.

[5] Bai H., Cheng R. (2024). Generalized population-based training for hyperparameter optimization in reinforcement learning. IEEE Trans. Emerg Top. Comput. Intell..

[6] Li D., Zeng Z. (2023). CRNet: a fast continual learning framework with random theory. IEEE Trans. Pattern Anal. Mach. Intell..

[7] Costa D.G., Silva I., Medeiros M., Bittencourt J.C.N., Andrade M. (2024). A method to promote safe cycling powered by large language models and AI agents. MethodsX.

[8] Buitrago-Esquinas E.M., Puig-Cabrera M., Santos J.A.C., Custódio-Santos M., Yñiguez-Ovando R. (2024). Developing a hetero-intelligence methodological framework for sustainable policy-making based on the assessment of large language models. MethodsX.

[9] Long S., Tan J., Mao B., Tang F., Li Y., Zhao M., Kato N. (2025). A survey on intelligent network operations and performance optimization based on large language models. IEEE Commun. Surv. Tutor..

[10] Jin Q., Wang Z., Floudas C.S., Chen F., Gong C., Bracken-Clarke D., Xue E., Yang Y., Sun J., Lu Z. (2024). Matching patients to clinical trials with large language models. Nat. Commun..

[11] Chen M., Wang T.-C. (2025). HyperPlace: harnessing a large language model for efficient hyperparameter optimization in GPU-accelerated VLSI placement. ACM Trans. Des. Autom. Electron. Syst..

[12] X. Yuan, Z. Yang, Z. Huang, Y. Wang, S. Fan, Y. Ju, J. Zhao, K. Liu, Exploiting contextual knowledge in LLMs through V-usable information based layer enhancement, in: Proc. 63rd Annu. Meet. Assoc. Comput. Linguist. (Vol. 1: Long Papers), 2025, pp. 31726–31741. doi:10.18653/v1/2025.acl-long.1531.

[13] Li D. (2023). Proc. Annu. Meet. Assoc. Comput. Linguist..

[14] Chen Y., Zhang Z., Han X., Xiao C., Liu Z., Chen C., Li K., Yang T., Sun M. (2024). Proceedings of the 2024 Joint International Conference on Computational Linguistics and Language Resources and Evaluation (LREC-COLING 2024).

[15] Park B.-J., Yong S.-J., Hwang H.-S., Moon I.-Y. (2025). Optimizing agent behavior in the MiniGrid environment using reinforcement learning based on large language models. Appl. Sci..

[16] Chen D., Huang Y. (2025). Integrating reinforcement learning and large language models for crop production process management optimization and control through a new knowledge-based deep learning paradigm. Comput. Electron. Agric..

[17] Ali Shahid A. (2025). Proc. IEEE Int. Conf. Autom. Sci. Eng..

[18] Zhang X., Li M., Yamane S. (2021). Proc. 2021 IEEE 10th Glob. Conf. Consum. Electron. GCCE 2021.

[19] Surrey D., Sharpe R.E., Gorniak R.J.T., Nazarian L.N., Rao V.M., Flanders A.E. (2013). QRSE: a novel metric for the evaluation of trainee radiologist reporting skills. J. Digit. Imaging.

[20] Chiu L.Z.F., Salem G.J. (2010). Time series analysis: evaluating performance trends within resistance exercise sessions. J. Strength Cond. Res..

